# The Effect of a Corrosion Inhibitor on the Rehabilitation of Reinforced Concrete Containing Sea Sand and Seawater

**DOI:** 10.3390/ma13061480

**Published:** 2020-03-24

**Authors:** Chonggen Pan, Xu Li, Jianghong Mao

**Affiliations:** 1 Ningbo institute of Technology, Zhejiang University, Ningbo 315100, China; panchonggen@zju.edu.cn (C.P.); 18764806631@163.com (X.L.); 2 Department of Civil Engineering, Shanghai University, Shanghai 200000, China; 3 Ningbo Research Institute, Zhejiang University, Ningbo 315100, China

**Keywords:** reinforced concrete, sea sand, durability, inhibitor, bidirectional electromigration(BIEM)

## Abstract

Concrete made with sea sand and seawater is rich in chlorine ions which are the main factors that induce corrosion of the reinforcement. In this study, an innovative method to rehabilitate reinforcement is presented; the concentrations of chloride ions and the corrosion inhibitor in concrete were measured. Electrochemical chloride extraction (ECE) was applied as a control experiment via using saturated Ca(OH)2 solution as an external electrolyte. Bidirectional electromigration (BIEM)technology combined with the corrosion inhibitor could not only remove the chloride ions but also protect the steel bar in concrete, and animidazoline inhibitor mixed in concrete is more effective than thetriethylenetetramine inhibitor due to the specific molecular structure. It was found that the optimum ratio of N/Cl reached the maximum value 3.3, when the concentration of inhibitor was 1. Meanwhile, the experimental results also revealed that the corrosion inhibitor and chloride ion concentrations reached necessary levels on the surface of the steel, and the corrosion inhibitor migrated effectively. Overall, the contents of imidazoline and triethylenetetramine inhibitor in seawater concrete are0.75% and 1%, respectively. The results demonstrate that the addition of the corrosion inhibitor and the application of bidirectional electromigration would effectively improve the durability of reinforced concrete containing sea sand and seawater.

## 1. Introduction

The last decade has witnessed an increasing demand forsand in the construction sector. However, there exists a short fall of river sand supplies, which has triggered a search for alternative sand resources. One possible alternative is desalinated sea sand, which, compared to river sand, offers several advantages: it has a lower mud content, harder particles and better gradation. Sea sand was first used in Great Britain in concrete structures [1,2]. Besides, it accounted for 12.2% of fine aggregates used in Japanese concrete in 2011; more than 90% of coastal projects used sea sand in concrete [3]. The Netherlands has also utilized a large amount of sea sand in non-stressed structural components [4]. In spite of this, sea sand and seawater contain chlorine salt and other erosive ions, which can potentially cause steel corrosion and adversely affect the durability of concrete structures [5,6,7]. For instance, the use of unqualified sea sand in Korea has caused corrosion and cracking problems in many buildings [8]. To ensure the durability of sea-sand concrete meets standards, it is necessary to devise specifications for the application of sea sand in concrete [9]. However, it remains impossible to have complete compliance of construction projects with standards due to the shortage of materials, and serious issues may occur in “sea-sand buildings” that affect the safety of people and the property. Therefore, it is of great importance to guarantee the durability and prolong the service lives of sea sand and seawater concrete buildings.

Many studies have been carried out on the durability of sea sand and seawater concrete. Some of them focused on developing different types of inhibitors. Tatematsua [10] synthesized a salt absorbent that can absorb excess chloride ions to prevent corrosion of steel bars in sea sand concrete. Following seven years of exposure, the corrosion inhibitors were found to observably inhibit steel corrosion in sea sand concrete. Hama [11] used calcium nitrate mixed with steel corrosion inhibitors, along with other substances, and demonstrated that combining a corrosion inhibitor and dispersant improved sea-sand concrete durability. Jamil [12] studied an alcohol amine corrosion inhibitor and dispersant, and found they formed a protective film on the steel surface, which significantly improved the charge transfer resistance of the steel bars and steel surface membrane resistance in sea-sand concrete. However, they did not fundamentally aim to eliminate the harmful media Cl-in sea sand and seawater concrete. Based on the principle of movement of electrochemical electricity, chloride removing technology is an important method of securing the durability of concrete. Eliminating the chloride ion within concrete mainly includes electrochemical dechlorination, corrosion resistance electro-osmosis and bidirectional electromigration technology. Electrochemical dechlorination [13,14] is the removal of chloride ions from concrete by applying an electric field. Orellan [15] demonstrated the efficiency of electrochemical removal of chlorides at a 1–10 A/m^2^ current density, when the concentration of chlorides could reach 50%–90% on the surface of the reinforcement embedded in the concrete. Ihekwaba [16] researched the electrochemical dechlorination which eliminated most of the chloride ions in the cylinder specimens. Electrochemical dechlorination was able to remove chloride ions from the surface of reinforcement; however, it would not protect steel reinforcement itself. Therefore, electro-osmotic [17] technology was developed to use electrochemical methods to transfer a corrosion inhibitor to the surface of a steel reinforcement to prevent corrosion. Holloway [18] confirmed the potential of electric fields in transferring corrosion inhibitors in concrete. Sanchez’s [19] research also proved that alkylamine corrosion-inhibiting agents can migrate to the surface of the reinforcement for adsorption and protection under the action of an electric field. However, the introduction of an inhibitor does not reduce the concentration of chloride ions within concrete which could be a hidden danger in the future. Bidirectional electrochemical technology [20,21] was thereby developed to combine electrochemical chlorine removal and introduction of inhibitor, which has been successfully employed by Xu [22]. All of these studies focus on improving the durability of existing structures, but few studies have been done on the durability of newly built concrete structures using electrochemical methods.

At present, the durability of new concrete engineering is guaranteed by adding a corrosion inhibitor into the concrete. However, a large dose of inhibitor is required, and there are some common problems, such as slow penetration, lack of penetration depth and overall difficulty in getting the corrosion inhibitor and dispersant to reach the surface of the steel [23]. Organic compounds employed as corrosion inhibitors can adsorb on the metal surface through heteroatoms such as nitrogen, oxygen, sulfur and phosphorus, but the alcamines inhibitor has a weak electromigration ability and cannot migrate to the surface of steel effectively. Under highly alkaline conditions, the degree of ionization of alcamines inhibitor is limited and not enough to dissociate rust, which limits its inhibiting effect. Imidazoline inhibitors are a newly trend in the field of electromigration inhibitor research, reported to beeffective organic corrosion inhibitors [15,16,17]. A novel, low-in-toxicity and efficient water-based corrosion inhibitor can effectively inhibit metal corrosion and be used in neutral medium environments, and in acidic and alkaline conditions. Meanwhile, electrochemical methods can improve the migration efficiency of ions in concrete. Moreover, the incorporation of corrosion inhibitors into newly-built concrete structures can protect steel bars from the beginning of service, helping to resist corrosion by chloride ions and increase the durability of concrete structures. Durability is a problem which comes into play over the years and concernsthe life of the structure. In this study, the imidazoline inhibitor and thetriethylenetetramine inhibitor were combined with bidirectional electromigration technology, and then the effect on the durability of concrete containing sea sand and seawater; and the corrosion potential of steel bars, the chloride ion concentration and the content of corrosion inhibitor in concrete, were investigated.

## 2. Materials and Methods

### 2.1. Materials

Sulfoxide chloride (Cl2OS; relative density, 1.676), lauric acid (≥98%), NaHCO3 (density, 2.159 g/cm^3^), ether (C4H10O; relative density, 2.6), ethylenediamine (C2H8O; relative density, 0.9), triethylamine (C6H15N; density, 0.728 g/mL) and 1,3-dibromopropane (C3H6Br2; density, 1.989 g/mL) were provided by chemical reagent factory (Ningbo, China), and were AR analytical reagent grade.

### 2.2. Synthesis of the Imidazoline Inhibitor

**Figure 1:**
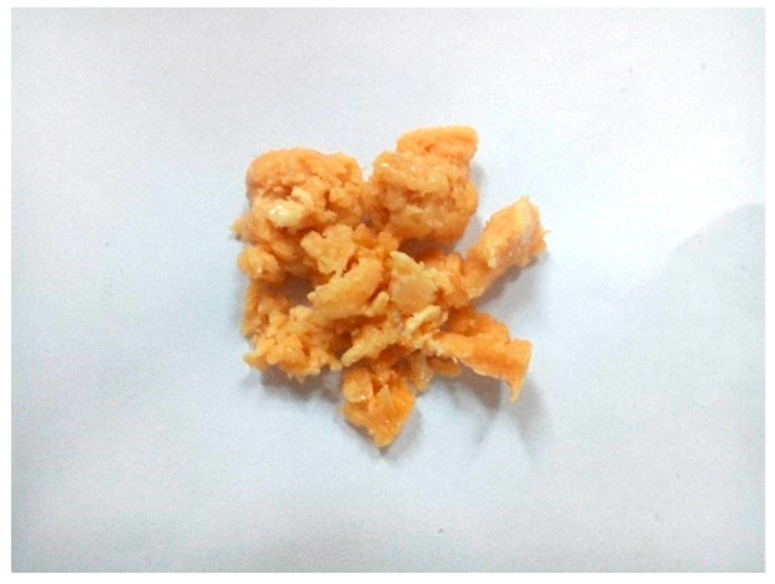
Imidazoline inhibitor.

In this study, a new imidazoline inhibitor was synthesized containing a symmetrical molecular structure with two imidazoline heterocycles and two alkyl long chains of lauric acid [24]. Synthesis of the imidazoline inhibitor is as follows [25]: Firstly, sulfoxide chloride was dropped into lauric acid and stirred for 1–5 h at 40–80 °C. Once the above reaction was completed, the lauroyl chloride was obtained, and then lauroyl chloride was added to the mixed solution of water containing NaHCO3 and ether before ethylenediamine was dripped. Ethylenediamine bislauroyl amide was obtained by stirring for 6 h at 10–40 °C, and then through filtering out the solid, washing and drying. Ethylenediamine bislauryl amide was mixed with phenoxyphosphatidyl diamide, which reacted at 100–250 °C for 5–60 min, and then cooled and heated at 50–100 °C for 10–60 min. While the monocyclic imidazoline was obtained after cooling, the monocyclic imidazoline lauric acid, 1,3-dibromopropane and triethylamine were dissolved in toluene and reacted for 10–25 h at 10–40 °C to obtain bicyclic imidazoline lauric acid. The synthesized imidazoline inhibitor was a light yellow solid (Figure 1).

### 2.3. Concrete Specimen

The reinforced concrete specimens had dimensions of 150 × 150 × 300 mm (height × width × length). Two light round steel bars (HPB235) with 10 mm diameters were embedded in the reinforced concrete specimens. The compressive strength of the concrete was C35, and the thickness of the protective layer was 40 mm, which according to the Chinese standard “Code for design of concrete structures (GB50010-2010),” and the environmental category of this study, is 3a. Ordinary Portland cement (OPC) was used with water-cement ratio of 0.48, and sea sand was used as fine aggregate. Sea sand and seawater were collected from Ningbo, China. The coordination of the reinforced concrete specimens is shown in Table 1.

### 2.4. Bidirectional Electromigration Technology

The basic principle underlying bidirectional electromigration technology was shown in our previous research [21,22]. During electrophoresis, the steel bar in the concrete is used as a cathode to connect to the negative pole of the DC power supply. the concrete specimen is wrapped with stainless steel mesh and acts as the anode which is connected to the positive pole of the DC power supply. The chloride ions inside the concrete can be removed by applying an electric field which drives the inhibitor to the surface of the steel bar. When the corrosion inhibitor reaches a critical concentration on the surface of the steel bar, a protective film would form on the surface of the steel bar, which insulates the surface of steel from the corrosive medium, thereby inhibiting the corrosion of steel.

**Figure 2:**
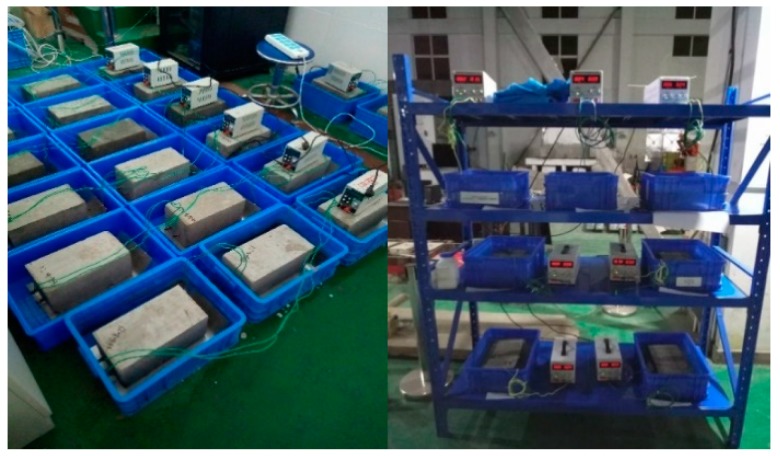
Experiment of bidirectional electromigration (BIEM).

Different amounts of imidazoline (IMDZ) and triethylenetetramine (TETA) were incorporated into concrete. The previous research found that the optimized amount of incorporated triethylenetetramine inhibitor was more than 2% of cementitious materials [24]. The corrosion inhibitor concentrations tested were 0.25%, 0.5%, 0.75%, 1%, 2% and 3% of the cementitious material. To investigate the effect of the electric field on the migration of the corrosion inhibitor, three concrete specimens were cast for each sample. The current density was set to 3 A/m2 and the electrification duration was 15 days [22]. In order to study the corrosion inhibition for the steel bars by the inhibitor, the electrochemical dechlorination (ECE) specimen was set as the reference sample and the electrification duration and current density were unchanged. The reinforced concrete specimens were removed from the mold after 2 days casting, and the electrification test was conducted with the bidirectional electromigration technique. The energization process of BIEM was shown in Figure 2.

### 2.5. Testing Methods of Reinforced Concrete

#### 2.5.1. Corrosion Potential of Rebar

After the power was turned on, the reinforced concrete specimens were taken out from device of electrochemical migration, and the concrete surface was cleaned. The specimens were then placed in a ventilated area for 72 h; after 72 h of standing, most of the residual electricity in the test specimen will dissipate, which will not affect the corrosion potential test results of the steel bars, and the performance of the concrete will also recover to more than 90%; then depolarization treatment was performed and the influence of residual electricity on the steel bars in the concrete after completion of the power supply was assessed for influence on the test results. The electrochemical refractometer 600 (Gamry, Warminster, PA, USA) was used to obtain the potentiometric polarization curve to investigate the effect of triethylenetetramine inhibitors on the corrosion resistance of concrete [25,26].

#### 2.5.2. Electrochemical Test

The electrochemical workstation was used for tests. A three-electrode system was adapted during tests. The electrochemical workstation started scanning at −70 mV with respect to the open circuit potential, with a scan interval of −70 to +70 mV, which is a weak polarized zone with scan rate being 0.15 mV/s. The data were analyzed with GamryEchem Analyst (Gamry, Warminster, PA, USA), an analysis software built into the electrochemical workstation Reference 600.

#### 2.5.3. Chloride Ion Concentration in Concrete

**Figure 3:**
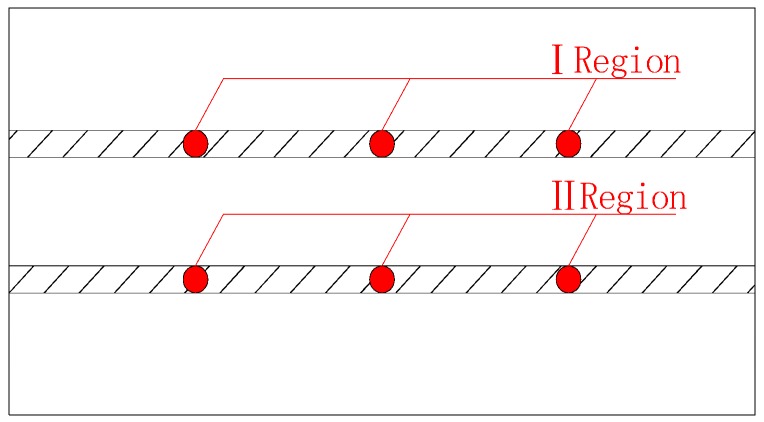
Position of concrete powder extracted area.

After the corrosion potential of the steel was tested, the concentration of residual chloride ions in the concrete after bidirectional electromigration was measured. The powder pick-up area was divided into areas I and II directly below the reinforcement. Three holes were created in each powder extraction area, as shown in Figure 3.

We used a drill with a diameter of 12 mm to take the powder which was extracted layer-by-layer every 5.0 mm until the surface of the steel bar was reached. After the powder was collected, 2.0 g of powder was accurately weighed and mixed with 20.0 g of deionized water. After soaking for 24 h, the Chloride-Meter DY-2501 precision ion meter was used to measure the chloride ion concentrations of the aqueous solutions [27,28].

#### 2.5.4. Content of Corrosion Inhibitor in Concrete

An additional hole was drilled in the reinforced concrete test piece. An impact drill with a diameter of 12 mm was used to collect powder at a depth of 10 mm along the direction of the protective layer. The concrete powder was oriented from the drill and passed through a sieve with a mesh size of 200. The sieved concrete powder was stored in a small sealed bag until the content of the corrosion inhibitor in the concrete being measured.

Since the corrosion-inhibiting group of the corrosion inhibitor contains nitrogen, the nitrogen element content, which indicates the corrosion inhibitor content in the concrete, was detected by an organic element analyzer. The concrete powder was stored in a small sealed bag wrapped with a tin capsule or silver sac and placed in an organic element analyzer (VarioMacro, Elementar, Langenselbold, Germany). A small amount of pure oxygen was added to help the sample fully burn. After combustion, the sample afurther catalytic oxidation-reduction, in which nitrogen was converted into detectable nitrogen. Finally, a thermal conductivity detector (TCD, Elementar, Langenselbold, Germany) was used to complete detection. The signal output by the TCD detector was directly converted into elemental content using integral calculations in a computer program.

## 3. Results and Discussion

### 3.1. Corrosion Potentialsof Steel Bars with Different Inhibitors

**Figure 4:**
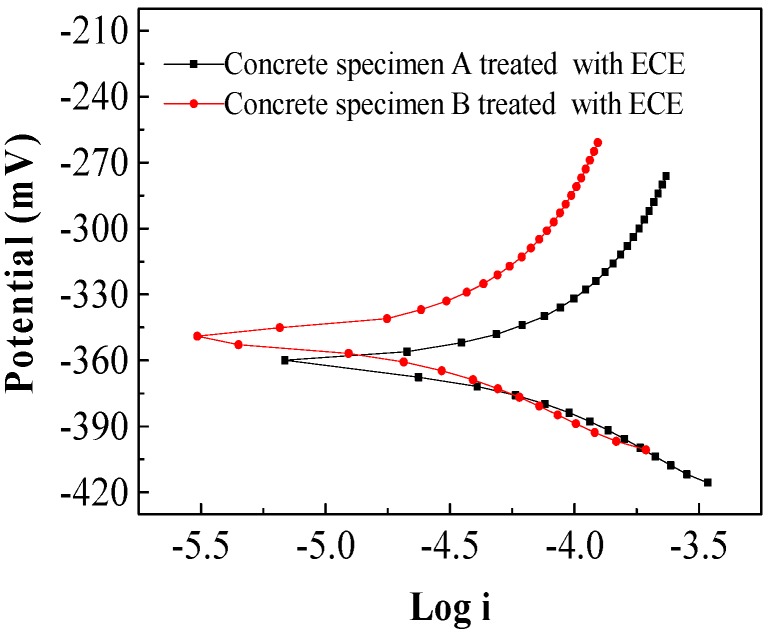
Polarization curves of a steel bar in concrete treated with electrochemical chloride extraction (ECE).

Figure 4 shows corrosion potential as a function of current. Normally, the corrosion potential values more negative than −250 mV can be regarded as due the fact that the reinforcement behaved with high corrosion risk according to the ASTM standard [28]. As indicated in Figure 4, the corrosion potentials of the steel bars in the concrete specimens A and B (with the same mixture proportions) were between −360 and −340 mV after treatment with electrochemical chloride extraction (ECE), which indicates an increasing rate of reinforcement corrosion risk.

**Figure 5:**
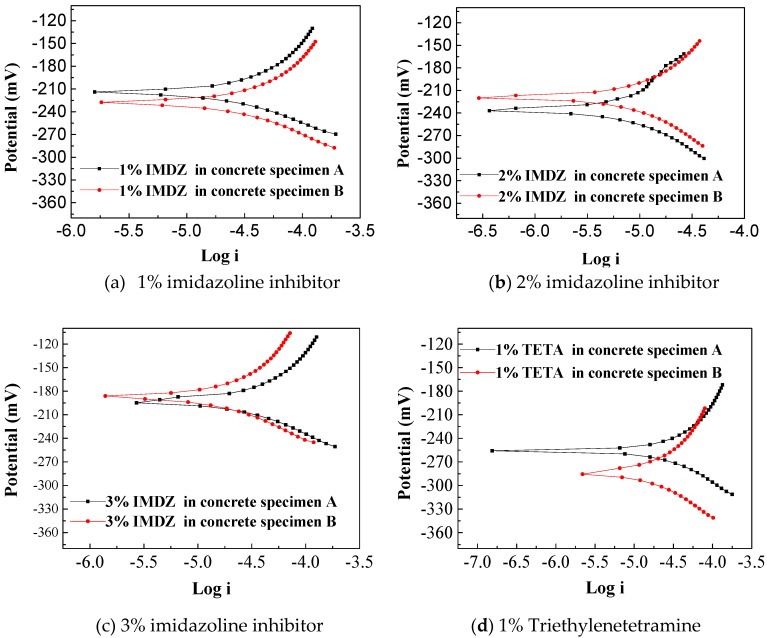

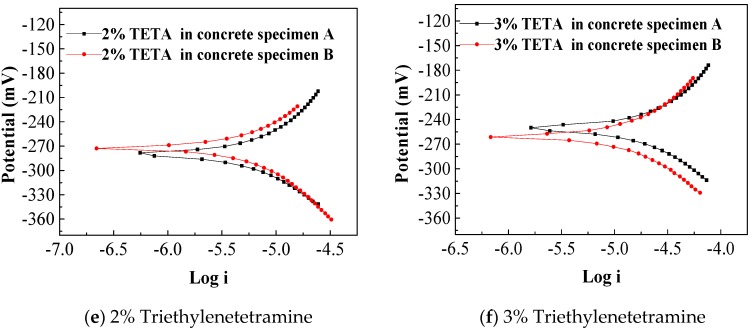
Polarization curves of a steel bar with a high concentration of inhibitor.

**Figure 6:**
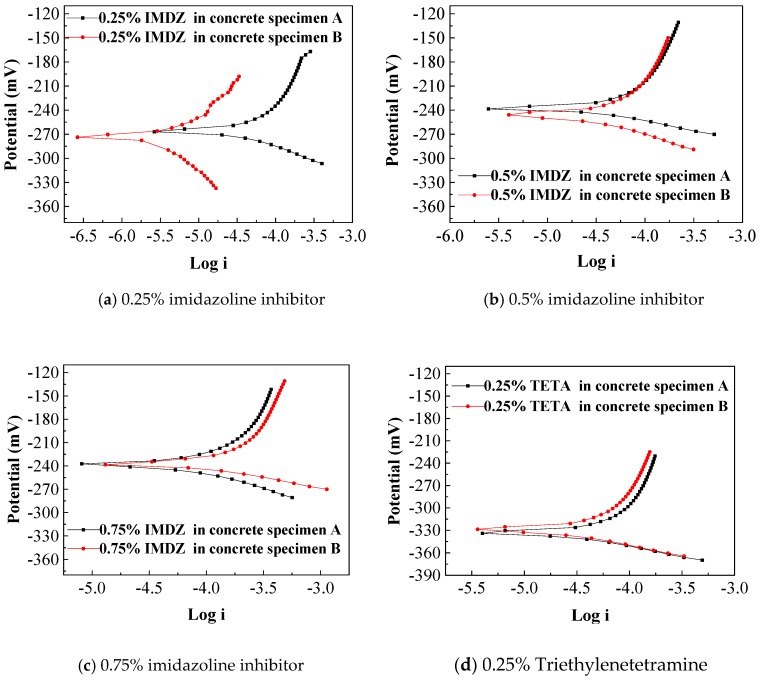

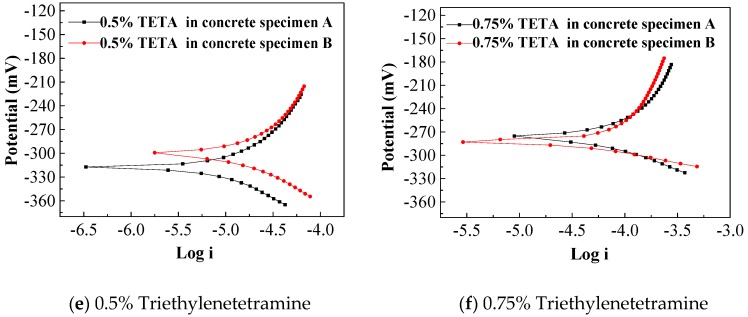
Polarization curves of a steel bar with a low concentration of inhibitor.

The corrosion potentials of the steel bars in the reinforced concrete specimens after bidirectional electromigration (BIEM) are shown in Figure 5 and Figure 6.

To analyze changes in the corrosion potentials of the steel bars, the corrosion potentials of the steel bars with different corrosion inhibitor contents are listed in Table 2.

As shown in Table 2, it can be seen that the corrosion potentials of the reinforced concrete specimens have a decline with increased concentration of triethylenetetramine inhibitor. The concrete specimens underwent BIEM and those containing inhibitor exhibited a positive shift compared to the corrosion potentials of ECE-treated concrete test pieces. For the concrete mixed with a high concentration of inhibitor from 1% to 3% (Figure 5), the corrosion potential of the steel bars decreased obviously, and if the concrete was mixed with a low concentration of inhibitor (0.25–0.75%, Figure 6), the reinforcement corrosion risk was still high, especially for triethylenetetramine group; the potential of steel is more than −250 mV [28]. When the concentration of imidazoline reached 0.5%, the corrosion potential of the steel bars was lower than −250 mV. Generally, the corrosion potential improvement of steel means a more extensive passivation region [29]. This shows that the corrosion inhibitor migrated to the steel surface under the application of the electric field and improved the corrosion potential of the steel bar and thus, which had a protective effect on the steel bar. As shown in above results, imidazoline inhibitor is more effective than triethylenetetramine inhibitor in concrete containing sea sand and seawater.

### 3.2. Chloride Concentration in Concrete

**Figure 7:**
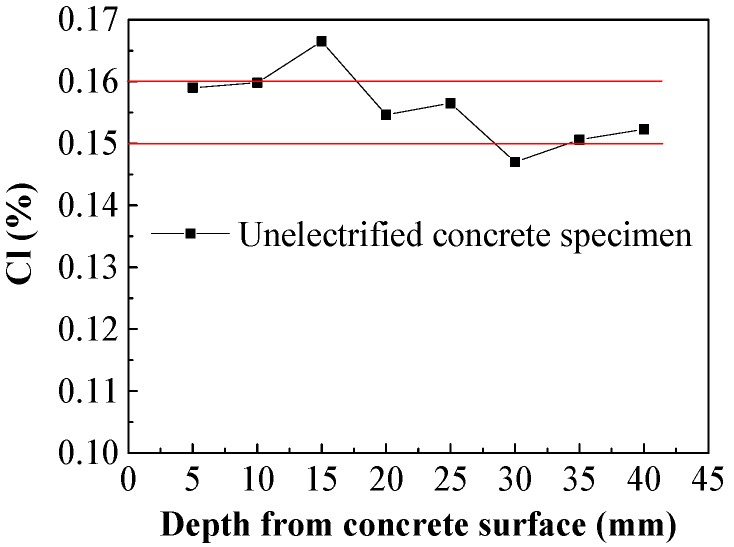
Initial chloride concentration in concrete.

As shown in Figure 7, the initial chloride ions distributed uniformly along the direction of concrete cover, and the concentration of chloride ions initially in the reinforced concrete specimens was around 0.15%–0.16% of the cementing material.

**Figure 8:**
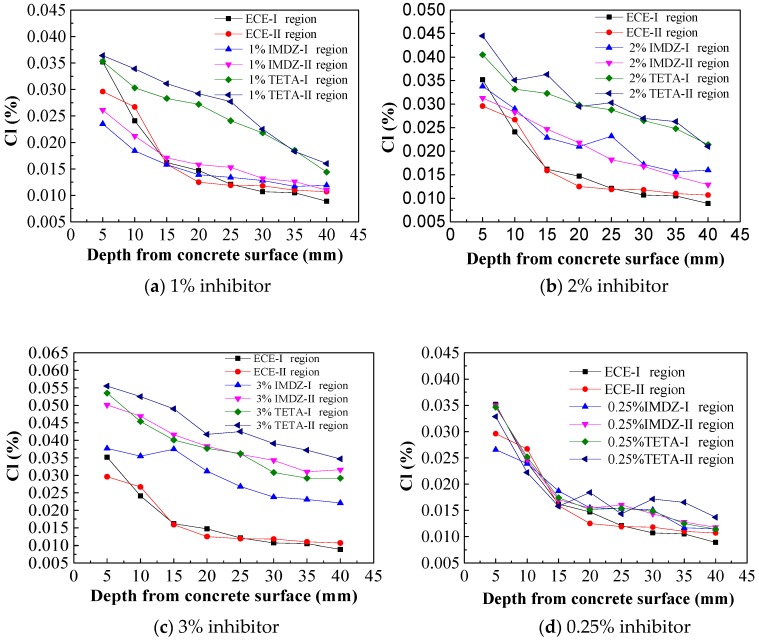

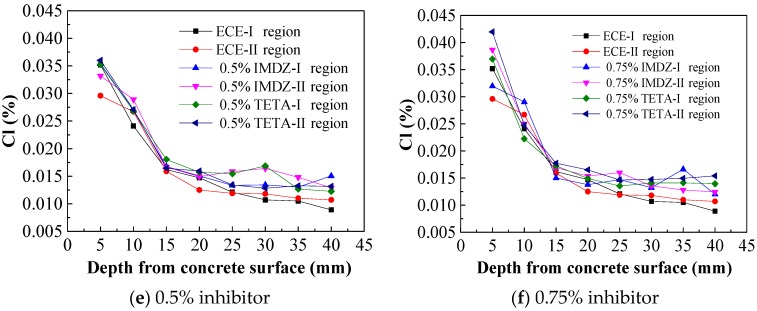
Chloride concentration in concrete.

The distributions of residual chloride ions in the concrete specimens after ECE and BIEM treatment are shown in Figure 8.

As shown in Figure 8, the residual chloride concentrations in the reinforced concrete specimens decreased along the thickness of the protective layer; that is, the residual chloride concentrations were highest on the surface of the concrete specimen and lowest in the steel bar. The concentrations of chloride ions in concrete specimens containing 0.25%, 0.5% or 0.75% inhibitor were slightly higher than that of ECE, which was 0.03%–0.04%. With the addition of 1%, 2% or 3% corrosion inhibitor, the chloride concentrations in the concrete specimen were obviously higher than those of samples with ECE treatment. This indicates that the addition of rust inhibitor influences the chloride ion discharge of concrete specimens, because to the chloride ions inside concrete can be removed by applying an electric field which drives the inhibitor to the surface of the steel bar in the opposite direction. With the addition of imidazoline and triethylenetetramine inhibitors, the residual chloride ion concentrations in concrete specimens increase with the addition of corrosion inhibitor; meanwhile, the removal efficiency of chloride ion decreases. With the same amount of inhibitor, the residual chlorine in concrete with imidazoline inhibitor is slightly lower than that of triethylenetetramine, and the removal efficiency of chloride ion is also higher.

### 3.3. Amount of Internal Resistance Corrosion Inhibitor in Concrete

**Figure 9:**
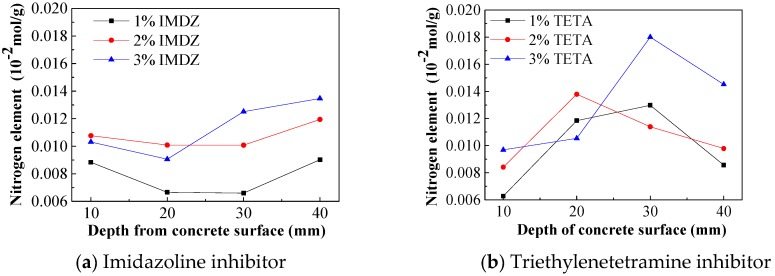

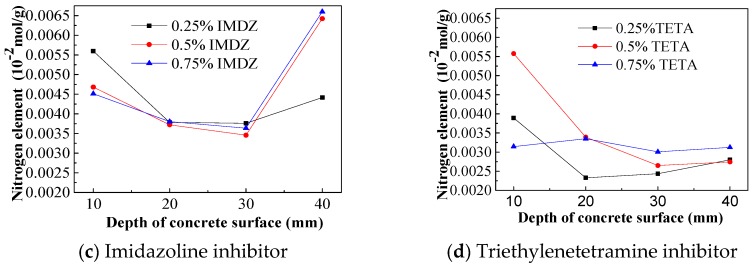
Content of corrosion inhibitor in depth of concrete surface.

The content of nitrogen in concrete powder was determined using an organic element analyzer; thus, the content of corrosion inhibitor in concrete was obtained. The content of corrosion inhibitor in concrete specimens is shown in Figure 9.

As shown in Figure 9a, the content of imidazoline inhibitor in concrete decreases first and then increases. The content of inhibitor in surface and steel bar position of concrete are higher than for the middle position. While the imidazoline inhibitor was mixed at 1%, 2% or 3%, the content of inhibitor in the steel bar position was 0.009×10^−2^, 0.012×10^−2^ or 0.0145×10^−2^ mol/g, respectively. In Figure 9b, it can be seen that the content of triethylenetetramine inhibitor in concrete increases first and then decreases with the highest content of corrosion inhibitor being in the middle of concrete. While the triethylenetetramine inhibitor was mixed at 1%, 2% or 3%, the content of inhibitor in the steel bar position was 0.0086×10^−2^, 0.0098×10^−2^ or 0.0145×10^−2^ mol/g, respectively. The content of triethylenetetramine inhibitor in the middle of concrete specimen is higher than that of imidazoline inhibitor, which can be learned from the distribution of residual chloride ion in concrete. The reason is that the dissociation degree of triethylenetetramine is limited in high alkali environment, and amounts of non-dissociated triethylenetetramine inhibitor accumulate in the middle of concrete specimens, which also affects chloride ion migration [30]. The concrete mixed with a low concentration of corrosion inhibitor is shown in Figure 9c,d; the trends of content of imidazoline and triethylenetetramine inhibitor are consistent in concrete. With the same dosage in concrete, the content of imidazoline is higher than that of triethylenetetramine, which means that imidazoline does better corrosion inhibition of the steel bar, and it also conducive to the durability of concrete.

It can be seen that the content of triethylenetetramine inhibitor decreases with the depth of concrete in the outer 20 mm, while it slightly increases with the concrete depth in the 30–40 mm. As seen in Figure 9b, for the concrete inner corrosion inhibitor contents of 1%, 2% and 3%, triethylenetetramine inhibitor generally increased before decreasing, with the highest content of corrosion inhibitor being in the middle position of concrete, indicating that the degree of dissociation of triethylenetetramine in a high alkali environment is limited [30], and triethylenetetramine does not dissociate in concrete—the central part of the specimen formed and accumulated.

### 3.4. The Influence of Inhibitor on the Corrosion Potentialsof the Steel Bars

**Figure 10:**
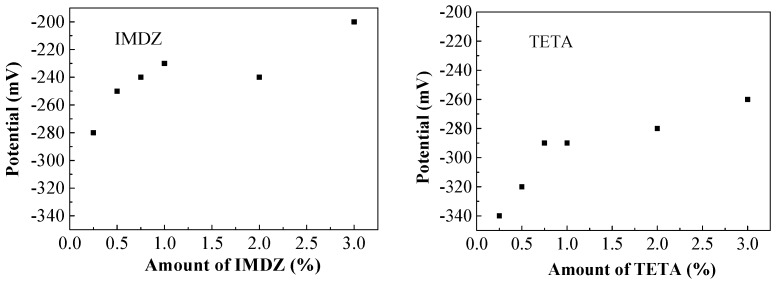
Corrosion potentials of the steel bars.

In order to investigate the influence of the amount of inhibitor on the corrosion potentials of steel bars, the relationship between the corrosion potential and the amount of inhibitor is presented in Figure 10.

**Figure 11:**
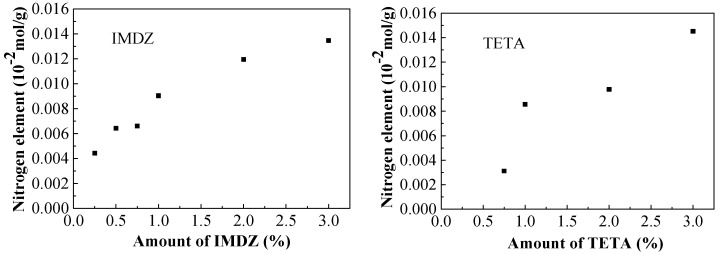
Nitrogen’s content.

It can be seen from Figure 10 that the corrosion potential increases significantly from 0.25% to 1% of corrosion inhibitor, while the growth rate of corrosion potential slows down when the corrosion inhibitor is above 1%. This indicates that the inhibitor has a positive influence on the corrosion potential and the content of 1% corrosion inhibitor is a critical value for the corrosion potential of steel rebar in sea water and sea sand, and the corrosion potential of reinforcing bars in concrete cannot be significantly increased by adding a large amount of rust inhibitor. As shown in Figure 11, from the relationship of the inhibitor content near the steel bar and the amount of mixed-in inhibitor, the nitrogen element content increases significantly from 0.25% to 1% of corrosion inhibitor, while the growth rate of nitrogen element content slows down when the corrosion inhibitor is above 1%. The results of Figure 10 and Figure 11 indicate a large amount of inhibitor in concrete specimens affects the discharge of chloride ions, which is not conducive to the durability of concrete. Therefore, considering the corrosion potential of steel bars, residual chloride ion concentration and the content of corrosion inhibitor in concrete, the imidazoline is more effective than the triethylenetetramine inhibitor; the specific molecular structure of imidazoline has a better migration and corrosion resistance. In order to ensure the durability of seawater concrete, the effective contents of imidazoline inhibitor and triethylenetetramine inhibitor are 0.75% and 1%, respectively.

### 3.5. Effects of Inhibitorson the Chloride Extraction

**Figure 12:**
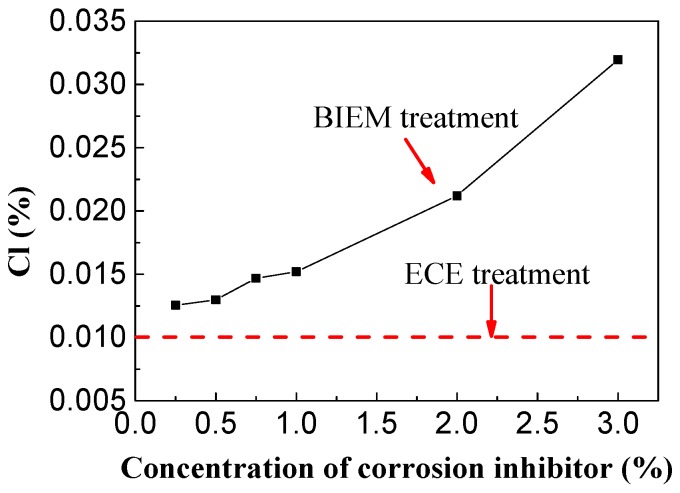
Relationship between corrosion inhibitor content and chloride ion concentration.

The chloride ion concentrations on the steel surface of the concrete specimens containing different concentrations of triethylenetetramine inhibitor were analyzed and the effect of the corrosion inhibitor concentration on the chloride extraction was studied, as shown in Figure 12.

It can be seen from Figure 12 that as the corrosion inhibitor increased, the concentration of residual chloride ions in the steel bar increased and the removal efficiency of chloride ions decreased, indicating chloride ion removal from concrete is affected by large doses of inhibitor. There was no obvious difference in the concentrations of residual chloride in the steel bar when 0.25%–1% triethylenetetramine inhibitor was used. However, chloride ion accumulation was increasingly serious with the addition of corrosion inhibitor, and the concentration of chloride ion in the steel bar with 3% corrosion inhibitor was high. Compared with electrochemical chlorination extraction (ECE), the chloride ion concentration in the sample was 226% higher. The accumulation of chloride ions in the steel bars may be due to the large amount of corrosion inhibitor accumulation and blockage of pores [31,32] in the concrete, thereby influencing chloride migration. The accumulation of chloride ions on the surface of steel bars would influence the long-term durability of concrete structures. Therefore, the concentration of triethylenetetramine corrosion inhibitor in concrete containing sea sand and sea water should be lower than 1%.

### 3.6. Influence of Corrosion Inhibitor Concentration on the Surface of a Steel Bar

**Figure 13:**
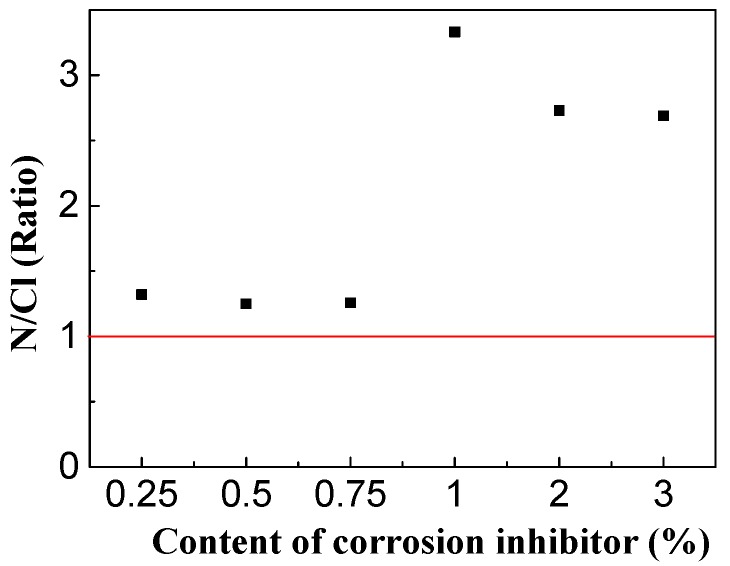
Corrosion inhibitor and Cl-content.

The respective amounts of corrosion inhibitor and chloride ions on the surfaces of the steel bars in concrete specimens were synthetically analyzed, and the effect of the corrosion inhibitor concentration on the amount of N/Cl^−^ on the steel surface was studied, as shown in Figure 13.

Previous study has shown that when the concentration of corrosion inhibitor near the steel bar is low, the corrosion inhibitor is not effective for protection of concrete [33]. For the triethylenetetramine inhibitor used, only when the concentration of the inhibitor in the pore solution was higher than the concentration of chloride, could the corrosion resistance be increased [34]. From Figure 13, it can be seen that the ratio of the N/Cl in concrete was more than 1 under different inhibitor concentrations, indicating the inhibitor content was higher than the concentration of chloride ions. The ratio of organic substances to harmful chloride ion concentration was optimized obviously for 1% inhibitor, reaching about 3.3. This shows that 1% corrosion inhibitor was the best concentration to improve the durability of sea sand and seawater concrete.

The presence of Cl- impedes the function of corrosion inhibitor even if the content of corrosion inhibitor is quite high. The high content of Cl-in the concrete is possibly attributable to the inhibition of the corrosion inhibitor from absorption of Cl-into concrete [35]. Thus, the protective effect of corrosion inhibitor in seawater or sea sand is obvious when the content of corrosion inhibitor reaches a critical value, which is 1% in this study.

## 4. Conclusions

In the above study, an amount of imidazoline and triethylenetetramine inhibitor were mixed into the concrete specimens which contained sea sand and seawater, and bidirectional electromigration (BIEM) technology was used. The effect of corrosion inhibitor on the durability of concrete was as follows:

(1) The corrosion potential showed that the imidazoline and triethylenetetramine inhibitor migrated to the steel surface under the application of BIEM, and improved the corrosion potential of the steel bar, and thus, had a protective effect on the steel bar. When the concentration of imidazoline reached 0.5%, the corrosion potential of the steel bars was lower than −250 mV.
(2) After BIEM treatment, the residual chloride ion concentrations in the reinforced concrete specimens decreased along the thickness of the protective layer. When the corrosion inhibitor content was high, the chloride ion concentrations in the samples containing the corrosion inhibitor were higher than those of electrochemical sample. With the addition of corrosion inhibitor, the chloride concentrations in the concrete specimen were obviously higher than those of samples with ECE treatment.
(3) When the inhibitor content was low, the content of corrosion inhibitor in the reinforced concrete specimens decreased along the concrete protective layer initially and then increased. When the inhibitor content was large, the corrosion inhibitor content in the reinforced concrete specimens was protected along the concrete. The tendency of the layer direction increased first, before decreasing due to the limited dissociation of triethylenetetramine in the high-alkali environment, where some undissociated triethylenetetramine accumulated in the center of the concrete specimen.
(4) This study showed the corrosion inhibitor can enhance the durability of the concrete containing sea sand and seawater. If the durability index of concrete becomes seriously degraded, it can continue to be energized to improve the durability of the concrete.

## Figures and Tables

**Table 1:** Mixture proportions.

Strength Grade	W/C	Cement Mark	Water (kg/m^3^)	Cement (kg/m^3^)	Sand (kg/m^3^)	Gravel (kg/m^3^)
C35	0.48	42.5	220	457.6	577.6	1072.6

**Table 2:** Corrosion potentials of steel bars.

Type of Inhibitor	Contents (%)	Corrosion Potential(mV)
Triethylenetetramine	0.25	−340~−320
	0.5	−320~−300
	0.75	−290~−270
	1	−290~−250
	2	−280~−270
	3	−260~−240
Imidazoline	0.25	−280~−260
	0.5	−250~−240
	0.75	−240~−230
	1	−230~−210
	2	−240~−220
	3	−200~−180
Ca(OH)_2_		−360~−340
